# Arginine methylation of SARS-Cov-2 nucleocapsid protein regulates RNA binding, its ability to suppress stress granule formation, and viral replication

**DOI:** 10.1016/j.jbc.2021.100821

**Published:** 2021-05-23

**Authors:** Ting Cai, Zhenbao Yu, Zhen Wang, Chen Liang, Stéphane Richard

**Affiliations:** 1 Segal Cancer Center, Lady Davis Institute for Medical Research and Gerald Bronfman Department of Oncology and Departments of Biochemistry, Human Genetics and Medicine, McGill University, Montréal, Québec, Canada; 2 McGill Centre for Viral Diseases, Lady Davis Institute for Medical Research and Department of Medicine, Department of Microbiology and Immunology, McGill University, Montréal, Québec, Canada

**Keywords:** SARS-CoV-2, type I PRMT inhibitor, nucleocapsid (N) protein, arginine methylation, PRMT1, RGG/RG motif, RNA binding, condensate, stress granules, AP, affinity-purification, CTD, C-terminal dimerization domain, DMSO, dimethylsulfoxide, FBS, fetal bovine serum, Flag-N, Flag-epitope N protein, GSK-3, glycogen synthase kinase 3, hnRNPA1, heterogeneous nuclear ribonucleoprotein A1, MERS-CoV, Middle East respiratory syndrome coronavirus, N, nucleocapsid, NTD, N-terminal RNA-binding domain, PAR-CLIP, photoactivatable ribonucleoside–enhanced crosslinking and immunoprecipitation, PFA, paraformaldehyde, PRMTs, protein arginine methyltransferases, RIP, RNA immunoprecipitation, S, spike, SARS-CoV-2, severe acute respiratory syndrome coronavirus 2, SGs, stress granules, SMN, survival of motor neuron, SR, serine/arginine-rich, SRPK, SRSF protein kinase, RBP, RNA binding protein, TDRD3, Tudor domain-containing protein 3, vRNP, viral ribonucleoprotein

## Abstract

Viral proteins are known to be methylated by host protein arginine methyltransferases (PRMTs) necessary for the viral life cycle, but it remains unknown whether severe acute respiratory syndrome coronavirus 2 (SARS-CoV-2) proteins are methylated. Herein, we show that PRMT1 methylates SARS-CoV-2 nucleocapsid (N) protein at residues R95 and R177 within RGG/RG motifs, preferred PRMT target sequences. We confirmed arginine methylation of N protein by immunoblotting viral proteins extracted from SARS-CoV-2 virions isolated from cell culture. Type I PRMT inhibitor (MS023) or substitution of R95 or R177 with lysine inhibited interaction of N protein with the 5’-UTR of SARS-CoV-2 genomic RNA, a property required for viral packaging. We also defined the N protein interactome in HEK293 cells, which identified PRMT1 and many of its RGG/RG substrates, including the known interacting protein G3BP1 as well as other components of stress granules (SGs), which are part of the host antiviral response. Methylation of R95 regulated the ability of N protein to suppress the formation of SGs, as R95K substitution or MS023 treatment blocked N-mediated suppression of SGs. Also, the coexpression of methylarginine reader Tudor domain-containing protein 3 quenched N protein–mediated suppression of SGs in a dose-dependent manner. Finally, pretreatment of VeroE6 cells with MS023 significantly reduced SARS-CoV-2 replication. Because type I PRMT inhibitors are already undergoing clinical trials for cancer treatment, inhibiting arginine methylation to target the later stages of the viral life cycle such as viral genome packaging and assembly of virions may represent an additional therapeutic application of these drugs.

The COVID-19 pandemic is caused by severe acute respiratory syndrome coronavirus 2 (SARS-CoV-2), a virus that belongs to the family *Coronaviridae* of genus *Betacoronavirus* and has a positive-sense strand RNA genome of ∼30 kb (1). It contains two large overlapping ORFs (ORF1a and ORF1b) and encodes four structural proteins, namely spike (S), envelope, membrane, and nucleocapsid (N) proteins as well as nine accessory proteins (1). ORF1a and ORF1b are further processed to generate 16 nonstructural proteins (Nsp1–16). Among the viral proteins, N protein is the most abundant in the virions and is expressed at the highest levels in infected cells (2). Thus, its abundance, essential roles during infection, and immunogenic nature make the SARS-CoV-2 N protein a valuable target for developing new strategies to combat the COVID-19 pandemic (3, 4, 5).

N protein regulates different steps of the coronavirus life cycle (2). The primary role of betacoronavirus N protein is the packaging of the viral genome into helical ribonucleoprotein complexes (6). It is also involved in RNA synthesis with components of the replicase at early stages of infection (7, 8). Betacoronavirus N protein has two conserved and independently folded structural domains, called the N-terminal RNA-binding domain (NTD) and C-terminal dimerization domain (CTD) (4, 9, 10), separated by flexible intrinsically disordered regions at the N terminus, central serine/arginine-rich (SR) linker region, and C-terminal tail, respectively. The crystal structure of the SARS-CoV-2 NTD RNA-binding domain depicts a U-shaped β-platform containing five short β-strands and an extended hairpin, forming a palm and finger-like structure with a highly positively charged cleft (4, 11).

After viral infection, host cells generate stress granules (SGs) as an antiviral response to inhibit protein synthesis and induce innate immune signaling (12, 13). SARS-CoV N protein plays an important role in host–virus interaction and localizes to cytoplasmic SGs (14). The SG nucleating factor G3BP1 (15, 16) and other SG components were identified in the SARS-CoV-2 N protein interactome (5, 17), suggesting SARS-CoV-2 like SARS-CoV regulates SGs mainly through N protein. The SARS-CoV-2 N protein is able to form condensates with RNA *in vitro* (18, 19, 20, 21, 22, 23, 24, 25), in the cytoplasm of cells (20, 24) and partially colocalizes within arsenite-induced SGs (26). Several studies have shown that N protein sequesters G3BP1 and disassembles SGs (21, 26, 27, 28), likely as a means to suppress the host immune response to favor virus replication. Recent studies show that intrinsically disordered region 1 and NTD regulate N protein condensates affecting nucleic acid annealing and potentially implicated in viral packaging and assembly (20, 21, 24). The SR linker region is phosphorylated by SRSF protein kinase (SRPK) (29), glycogen synthase kinase 3 (GSK-3) (30), and cyclin-dependent kinase 1–GSK3 (18), influencing N protein condensates (18, 21). Besides phosphorylation, post-translational modifications that regulate N function are not known.

We identify that SARS-CoV-2 N protein contains five undefined and uncharacterized RGG/RG motifs. RGG/RG motifs are prevalent in RNA binding proteins (RBPs) and play key roles in mediating protein–protein and protein–RNA interactions (31, 32). The arginine residues located within the RGG/RG motifs are the preferred sites of methylation by protein arginine methyltransferases (PRMTs) (33). In mammals, there are nine PRMTs (PRMT1–9) that are classified into three types based on the methyl marks they produce: *N*^*G*^-monomethylarginine, asymmetric *N*^*G*^, *N*^*G*^-dimethylarginine, and symmetric *N*^*G*^, *N'*^*G*^ dimethylarginine (33). Methylarginines are bound by Tudor domains that are methylarginine readers (34). Arginine methylation regulates protein–protein interactions and protein–nucleic acid interactions to influence basic cellular processes, including transcription, RNA processing including pre-mRNA splicing, mRNA export, and mRNA translation, signaling transduction, and liquid–liquid phase separation (35, 36). Unlike lysine demethylation, dedicated arginine demethylases have not been identified (36). Many specific small-molecule inhibitors of PRMTs have been generated for cancer therapeutics (37, 38, 39, 40, 41, 42), and a few have entered clinical trials (for review, see (36)).

Arginine methylation is known to methylate host and viral proteins necessary for viral replication. For example, the arginine methylation of HIV Tat protein decreases its transactivation function (43). The inhibition of PRMT5 prevents host heterogeneous nuclear ribonucleoprotein A1 (hnRNPA1) RGG/RG motif methylation and inhibits HIV-1 and human T-cell lymphotropic virus type-1 internal ribosome entry sites function (44). Moreover, PRMT5 methylates hepatitis B virus core protein within its C-terminal arginine-rich domain to regulate its cellular localization (45). Arginine methylation of prototype foamy virus Gag in its glycine–arginine–rich box by PRMT1 regulates its nucleolar localization during replication (46).

In the present study, we report that PRMT1 methylates SARS-CoV-2 N protein within its RGG/RG motifs to regulate the RNA-binding activity of the N protein toward its 5’-UTR genomic RNA. Moreover, arginine methylation modulates the role of N protein to inhibit SG formation. Our findings show for the first time that inhibition of type I PRMTs decreased SARS-CoV N methylation within virions and that arginine methylation is required for viral production.

## Results

### SARS-CoV-2 N protein is methylated by PRMT1

**Figure 1:**
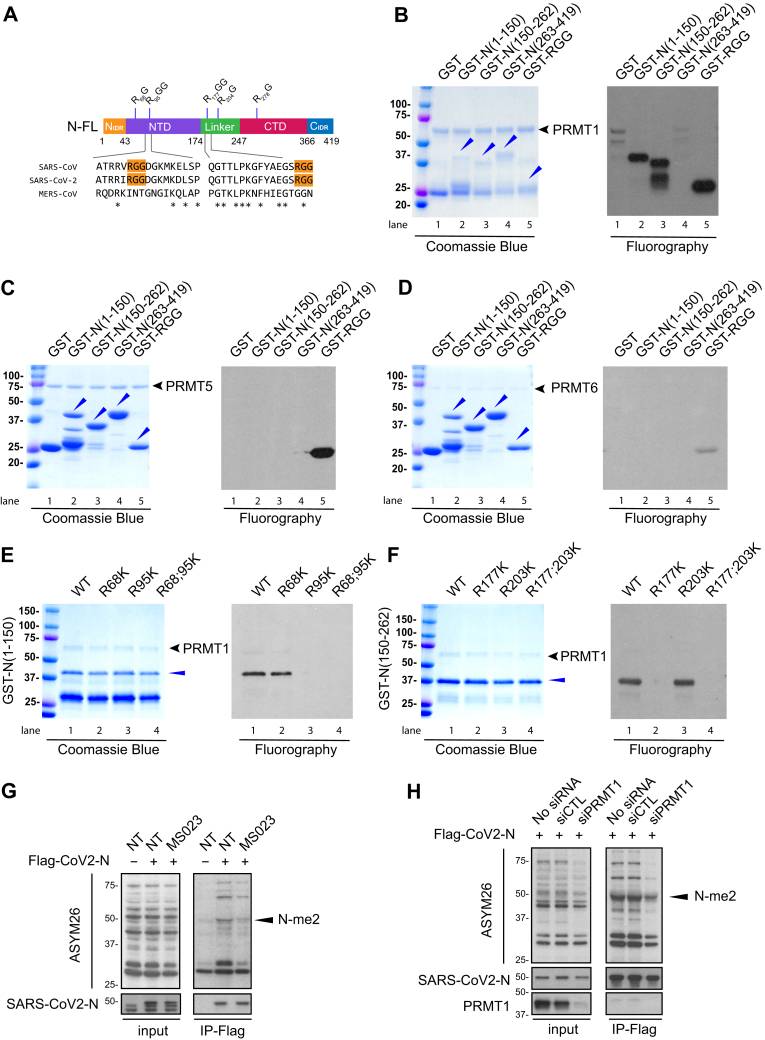
**R95 and R177 within SARS-CoV-2 N RGG/RG motifs are methylated by PRMT1.***A*, schematic diagram of N protein with its N-terminal domain (NTD) and C-terminal domain (CTD) and its N_IDR_ and C_IDR_ for N- and C-terminal intrinsic disordered regions and finally the linker region between NTD and CTD known for its SR-rich sequences. Note R95GG and R177GG are conserved in SARS-CoV and SARS-CoV-2, but not MERS-CoV. *B*–*D*, recombinant GST–N protein fragments were subjected to *in vitro* methylation assays with recombinant (*B*) GST–PRMT1, (*C*) PRMT5/MEP50, and (*D*) GST–PRMT6. Coomassie Blue staining and fluorography images are presented. GST alone and GST–RGG were used as negative and positive controls, respectively. *Blue arrowheads* indicate the migration of the GST–N protein fragments. The migration of GST–PRMT1, PRMT5, and GST–PRMT6 is shown on the *right* with a *black arrow*. The molecular mass markers are shown in kDa on the *left*. *E* and *F*, GST–N protein fragments with arginine to lysine substitution were subjected to *in vitro* methylation assays. Coomassie Blue staining and fluorography images are presented. *G*, HEK293 cells were transfected with control (-) or Flag-N (+) expression vectors for 24 h and incubated with or without (NT) 1 μM MS023 for another 24 h. The cell lysates were subjected to immunoprecipitation with anti-Flag-M2 beads and immunoblotting with anti-asymmetrical dimethylarginine antibody ASYM26 (*upper panels*) and anti-SARS-CoV-2 N protein antibody (*lower panels*). The band of the asymmetrically dimethylated N protein (N-me2) is marked by a *black arrowhead* on the *right*. The molecular mass markers are shown in kDa on the *left*. *H*, HEK293 cells were transfected with siRNA targeting firefly luciferase (siCTL) or siPRMT1 for 24 h and subsequently transfected with Flag-N vector for another 24 h. The cell lysates were subjected to immunoprecipitation with anti-Flag-M2 beads and then immunoblotting with anti-SARS-CoV-2 N protein and anti-ASYM26 antibodies. The migration of the methylated N protein is indicated. Flag-N, Flag-epitope N protein; MERS-CoV, Middle East respiratory syndrome coronavirus; N, nucleocapsid; PRMTs, protein arginine methyltransferases; SARS-CoV-2, severe acute respiratory syndrome coronavirus 2.

We noted that the SARS-CoV-2 N protein harbors two RGG (Fig. 1*A*) and three RG motifs like SARS-CoV, but unlike Middle East respiratory syndrome coronavirus (MERS-CoV). As RGG/RG motifs are preferred sites of PRMT1, PRMT5, and PRMT6 (31), we tested whether they could be methylated. We first expressed and purified glutathione S-transferase (GST)-fusion proteins of the SARS-CoV-2 N protein fragments (GST-N 1–150, GST-N 150–262, and GST-N 263–419, Fig. 1*A*) and performed *in vitro* arginine methylation assays. Both the N-terminal fragment (amino acid residues 1–150) and the central region (amino acid residues 150–262) were arginine-methylated by PRMT1 (Fig. 1*B*). By contrast, the N protein fragments were not methylated by PRMT5 or PRMT6 (Fig. 1, *C* and *D*). We then substituted arginines in the RGG/RG motifs to lysines to identify the methylated sites and maintain the charge. Mutation of arginine 68 (R68K) in the N-terminal fragment had no significant effect on arginine methylation, whereas mutation of arginine 95 (R95K) completely abolished PRMT1 methylation (Fig. 1*E*), suggesting that R95 was the methylated residue in the GST-N 1 to 150 fragment. Similarly, mutation analysis identified R177 as the methylated residue in the central fragment (Fig. 1*F*). Taken together, R95 and R177 within the RGG/RG motifs of the SARS-CoV-2 N were methylated *in vitro* by PRMT1.

We then determined whether the SARS-CoV-2 N protein was methylated in cells. HEK293 cells were transfected with a plasmid expressing Flag-epitope N protein (Flag-N). The cells were lysed, and the N protein was immunoprecipitated with anti-Flag antibodies and its methylation detected by Western blotting using the ASYM26 antibody, which specifically recognizes asymmetrically dimethylated arginine residues within RGG/RG motifs. Importantly, the asymmetrical dimethylarginine methylation of the Flag-N (N-me2) was significantly reduced by treatment of the cells with the type I PRMT inhibitor MS023 (Fig. 1*G*) and transfection with siPRMT1 (Fig. 1*H*).

We next monitored patient data to identify modulation of PRMT1 expression during SARS-CoV-2 infection. Single-cell RNA sequencing analysis of nasopharyngeal and bronchial samples from 19 clinically well-characterized SARS-CoV-2 patients and five healthy controls was performed (47). Importantly, analysis of their data showed that PRMT1 was significantly upregulated in infected patients (Fig. S1). These data suggest PRMT1 may play a role during the SARS-CoV-2 life cycle.

### The SARS-CoV-2 N interactome defines a complex of RGG/RG proteins and PRMT1

**Figure 2:**
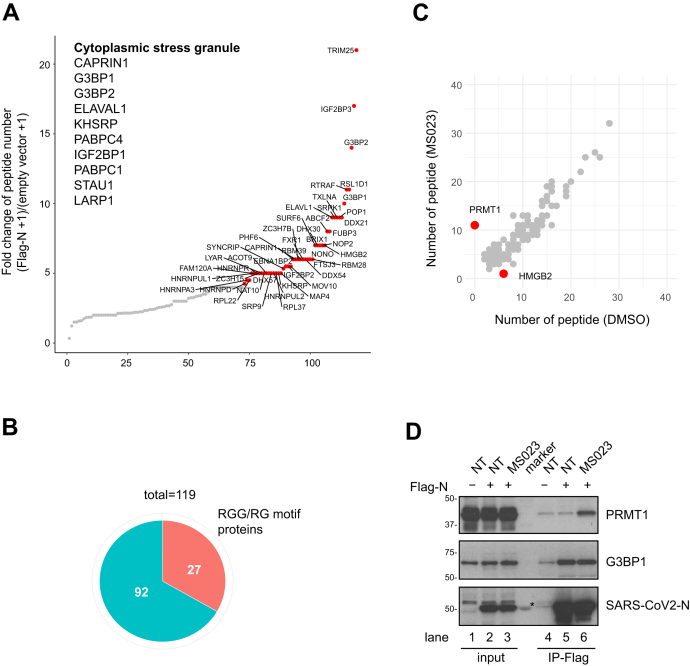
**N protein interactome with and without MS023: association with many RGG/RG proteins and PRMT1.** HEK293 cells were transfected with control or Flag-N, and the next day, Flag-N-transfected cells were subsequently treated with or without 1 μM MS023 for 24 h. Cell lysates were subjected to immunoprecipitation using anti-Flag-M2 beads. The bound proteins were identified by MS (*A*–*C*). *A*, interactors were ranked by fold change of unique peptides detected from Flag-N-transfected cells and control plasmid-transfected cells (Flag-N + 1)/(empty vector + 1). Proteins with FC >4 are highlighted in *red*. Immunoprecipitated proteins known to be localized in stress granule are listed. *B*, correlation analysis between MS023 and DMSO-treated N protein interactome is shown. Proteins with a significant fold change (>3 or <3^−1^) after MS023 treatment are highlighted in *red*. *C*, the pie chart represents the number of RGG/RG motif containing proteins among N protein interactors. *D*, HEK293 cells were transfected with control (-) or Flag-N (+) and subsequentially treated with or without (NT) 1 μM MS023 for 24 h. Cell lysates were immunoprecipitated with anti-Flag antibodies, and the associated proteins separated by SDS-PAGE and immunoblotted with anti-PRMT1, anti-G3BP1, and anti-SARS-CoV-2 N antibodies. The *asterisk* denotes nonspecific recognition of a molecular mass marker protein. DMSO, dimethylsulfoxide; PRMTs, protein arginine methyltransferases; SARS-CoV-2, severe acute respiratory syndrome coronavirus 2.

We then performed MS analysis to identify SARS-CoV-2 N-interacting proteins in the absence or presence of MS023. Flag-tagged SARS-CoV-2 N protein was expressed in HEK293 cells and a pull-down performed using anti-Flag affinity resin. Co-purified cellular proteins were subsequently analyzed by affinity-purification (AP) LC-MS/MS. We identified 119 cellular proteins interacting with SARS-CoV-2 N protein (peptide count >2, fold change >2 between Flag-N and empty vector transfected, 0.1% false discovery rate, Fig. 2, *A* and *B*, Supplementary Dataset 1). Importantly, we identified several protein components of SGs such as G3BP1 and G3BP2 (Ras-GTPase–activating protein SH3 domain-binding protein 1 and 2) (48), and CAPRIN1 (Fig. 2*A*), in line with previous published AP-MS/MS studies (5, 17). Moreover, our MS analysis identified SRPK1 and GSK-3, known to phosphorylate N protein and regulate its localization to SGs (18, 21, 29). We also identified TRIM25 as a top hit in the N protein interactome. As a K63-linked ubiquitin ligase, TRIM25 mediates retinoic acid–inducible gene 1 ubiquitination and activates TLR/RLR signaling pathway in response to RNA virus infection. It is known that SARS-CoV N protein interacts with TRIM25 and inhibits TRIM25/retinoic acid–inducible gene 1 association (49), suggesting that SARS-CoV-2 N protein may play a similar role in antagonizing the host immune response.

We then performed biological process (Gene Ontology) analysis using the identified interaction partners to assess major cellular pathways. The top ten pathways enriched consisted of RNA metabolic processes (Fig. S2). Interestingly, many SARS-CoV-2 N-interacting proteins (27 of 119, Fig. 2*B*) contained multiple RGG/RG motifs including DEAD/DExH family of RNA helicases DDX21, DDX54, DHX30, DHX57, and hnRNPA1, A3, D, DL, G (RBMX), R, U, UL1, and UL2 (Table 1). Many of these N-interacting proteins, such as G3BP1 (50), FAM98A (51), FXR1 (52), hnRNPA1 (53), hnRNPUL1 (54), SYNCRIP (55), ILF3 (56), and SERBP1 (57) are known PRMT1 substrates.

**Table 1:** RGG/RG motif containing proteins within SARS-CoV-2 N interactome

ID	Name	RNA binding
Q14444	CAPRIN1	Yes
Q8TDD1	DDX54	Yes
Q6P158	DHX57	Yes
Q08211	DHX9	Yes
Q01844	EWSR1	Yes
Q9NZB2	FAM120A	Yes
Q8NCA5	FAM98A	Yes
P22087	FBL	Yes
P51114	FXR1	Yes
Q13283	G3BP1	Yes
Q9UN86	G3BP2	Yes
O14979	HNRNPDL	Yes
Q9BUJ2	HNRNPUL1	Yes
Q1KMD3	HNRNPUL2	Yes
Q14103	HNRNPD	Yes
O60506	SYNCRIP	Yes
O43390	HNRNPR	Yes
Q00839	HNRNPU	Yes
Q9NZI8	IGF2BP1	Yes
Q12905	ILF2	Yes
Q12906	ILF3	Yes
Q8NC51	SERBP1	Yes
Q99575	POP1	Yes
P38159	RBMX	Yes
P09651	HNRNPA1	Yes
P51991	HNRNPA3	Yes
P46783	RPS10	Yes

N protein interactome changed significantly by two proteins with MS023 treatment. Interaction between N protein and HMGB2 was lost, and interaction with PRMT1 was gained with MS023 treatment (Fig. 2*C*). HMGB2 is a paralog of HMBG1 shown to play a critical role in SARS-CoV-2 replication (58). Interestingly, the AP-MS/MS analysis identified 11 PRMT1 peptides covering 30% of the protein sequence in the Flag-N immunoprecipitation from MS023-treated cells and none in nontreated cells (Fig. 2*C*). These data are consistent with MS023 being noncompetitive with type I PRMT substrates (37). To confirm these interactions, HEK293 cells were transfected with Flag-N and treated with dimethylsulfoxide (DMSO) control or MS023. Cell extracts were prepared and immunoprecipitations performed with anti-Flag antibodies followed by SDS-PAGE and immunoblotted with anti-PRMT1, anti-G3BP1, or anti-SARS-CoV2 N protein antibodies. Indeed, Flag-N immunoprecipitations showed increased PRMT1 association with MS023 treatment and G3BP1 was observed in immunoprecipitations from treated and nontreated cells (Fig. 2*D*, compare lanes 5 and 6). These data confirm interactions between the N protein and G3BP1 and PRMT1.

### SARS-CoV-2 N prevents SG formation in an arginine methylation–dependent manner

**Figure 3:**
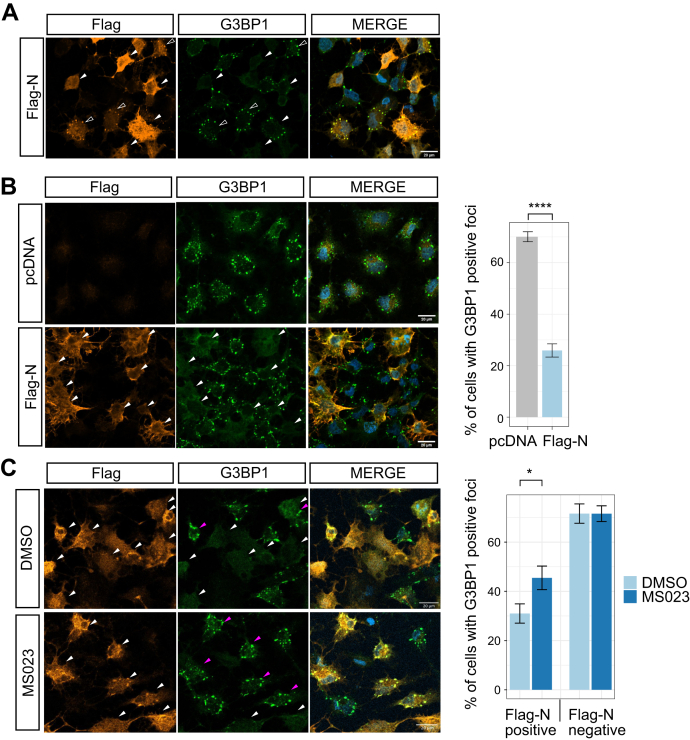
**SARS-CoV-2 N protein regulates G3BP1 stress granule formation in an arginine methylation-dependent manner.***A*, Huh-7 cells were transfected with control vector or Flag-N for 24 h and subsequently incubated with 1 mM sodium arsenite for 2 h. Cells were fixed with 4% PFA and coimmunostained with anti-Flag and anti-G3BP1 antibodies. A typical image is shown. The scale bar represents 20 μm. *Arrowheads* indicate FLAG-N-transfected cells, and the *empty arrowheads* indicate cells with FLAG-N and G3BP1 colocalization. *B*, Huh-7 cells were transfected with control vector or Flag-N for 24 h and subsequently incubated with 0.5 mM sodium arsenite for 1 h. Cells were fixed with 4% PFA and coimmunostained with anti-Flag and anti-G3BP1 antibodies. A typical image is shown. The scale bar represents 20 μm. *Arrowheads* indicate FLAG-N-transfected cells, and transfected cells with SGs (>5 G3BP1 foci) are highlighted in *magenta*. The percentage of cells harboring SGs (>5 G3BP1 foci) are quantified and shown in the bar plot on the *right*. n = 15 fields from three independent experiments are shown. Welch's *t* test. ∗∗∗∗*p* < 0.0001. *C*, Huh-7 cells were transfected with Flag-N overnight and treated with or without 5 μM MS023 for another 24 h. Then, the cells were incubated with 0.5 mM sodium arsenite for another hour and fixed with 4% PFA. Cells were coimmunostained with anti-Flag and anti-G3BP1 antibodies. A typical image is shown in the *left panel*. The scale bar represents 20 μm. *Arrowheads* indicate Flag-N-transfected cells, and transfected cells with SGs (>5 G3BP1 foci) are highlighted in *magenta*. The percentage of cells harboring SGs (>5 G3BP1 foci) was quantified in the transfected cells (Flag-N positive) and nontransfected cells (Flag-N negative), respectively, and shown in the bar plot on the *right*. Twenty fields from two independent experiments are shown. Welch's *t* test. ∗*p* < 0.05. Flag-N, Flag-epitope N protein; N, nucleocapsid; PFA, paraformaldehyde; SARS-CoV-2, severe acute respiratory syndrome coronavirus 2; SGs, stress granules.

**Figure 4:**
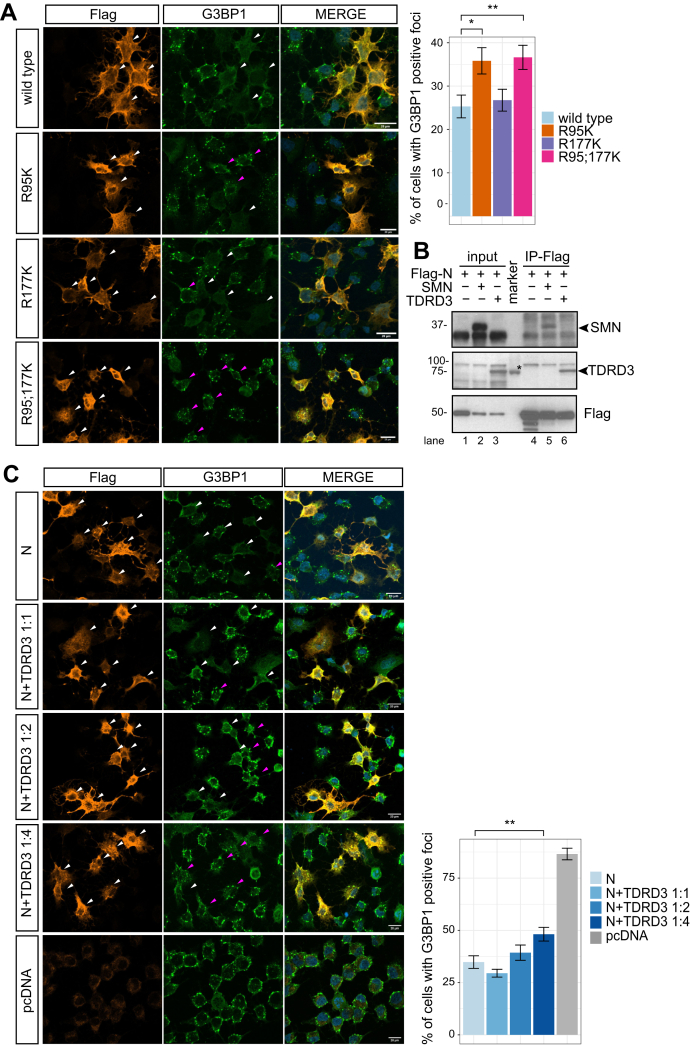
**R95 is required for N protein–regulating G3BP1 stress granule, and methylarginine reader protein TDRD3 is involved in this process.***A*, Huh-7 cells were transfected with Flag-N (WT) and its mutants for 24 h and subsequently incubated with 0.5 mM sodium arsenite for another hour. Cells were fixed with 4% PFA and coimmunostained with anti-Flag and anti-G3BP1 antibodies. A typical image is shown in the *left panel*. The scale bar represents 20 μm. *Arrowheads* indicate transfected cells, and transfected cells with SGs (>5 G3BP1 foci) are highlighted in *magenta*. The percentage of cells harboring SGs (>5 G3BP1 foci) in the Flag-N-positive cell group was quantified and shown in the bar plot on the *right*. n = 15 fields from three independent experiments are shown. Welch's *t* test. ∗*p* < 0.05, ∗∗*p* < 0.01. *B*, HEK293 cells were cotransfected with Flag-N and myc-SMN or myc-TDRD3. Twenty-four hours later, cell lysates were immunoprecipitated with anti-Flag antibodies and the associated proteins separated by SDS-PAGE and immunoblotted with anti-SMN, anti-TDRD3, and anti-Flag antibodies. The *asterisk* denotes nonspecific recognition of a molecular mass marker protein. *C*, Huh-7 cells were cotransfected with Flag-N and myc-TDRD3 with indicated plasmid ratios. Twenty-four hours later, cells were incubated with 0.5 mM sodium arsenite for another hour. Cells were fixed with 4% PFA and coimmunostained with anti-Flag and G3BP1 antibodies. A typical image is shown on the *left*. The scale bar represents 20 μm. *Arrowheads* indicate transfected cells, and transfected cells with SGs (>5 G3BP1 foci) are highlighted in *magenta*. The percentage of the transfected cells (Flag-N positive) harboring SGs (>5 G3BP1 foci) are quantified and shown in the bar plot on the *right*. n > 15 fields from two independent experiments are shown. Welch's *t* test. ∗∗*p* < 0.01. Flag-N, Flag-epitope N protein; PFA, paraformaldehyde; SGs, stress granules; SMN, survival of motor neuron; TDRD3, Tudor domain-containing protein 3.

SGs are frequently observed upon infection with DNA or RNA viruses, serving an antiviral function (12, 13). Recent studies reveal that SARS-CoV-2 N protein is associated with SGs and regulates their dynamics (21, 26, 27, 28). To investigate whether arginine methylation regulates the property of SARS-CoV-2 N protein to suppress SG formation and dynamics, we monitored SG formation using anti-G3BP1 antibodies in the hepatoma Huh-7, a cell line frequently used in the study of SARS-CoV-2. Huh-7 cells transfected with an empty plasmid (pcDNA) or a plasmid expressing Flag-N were treated with a mild dose of oxidative stress (0.5 mM sodium arsenite for 1 h) or a harsh dose (1 mM sodium arsenite for 2 h). At 1-μM sodium arsenite, we observed Flag-N colocalizing with G3BP1 in SGs (open arrowheads), and in some Flag-N–expressing cells, there was a reduction or absence of G3BP1 SGs (white arrowheads) (Fig. 3*A*), as reported recently (21, 26, 27, 28). Interestingly, at the mild doses of 0.5 mM sodium arsenite for 1 h, 25.89 ± 2.56% of Flag-N-transfected cells had G3BP1 SGs compared with 70.08 ± 1.93% in the pcDNA-transfected cells (Fig. 3*B*), suggesting that SARS-CoV-2 N protein suppresses G3BP1 SG formation. As regulating SGs formation is an important function for viral replication and host cell immune response (13), we focused our study on how arginine methylation was implicated in N protein–mediated SG suppression. Thus, all subsequent studies were performed with 0.5 mM sodium arsenite for 1 h to study N protein inhibition of G3BP1 SGs. Huh-7 cells transfected with Flag-N were treated with type I PRMT inhibitor MS023 or control DMSO before induction of SGs with sodium arsenite. Methyltransferase inhibition using MS023 significantly increased the presence of Flag-N–expressing cells with G3BP1 SGs (45.48 ± 4.79% *versus* 30.99 ± 3.92%), while no significant change was observed in the nontransfected cells with over >70% of the cells with SGs (Fig. 3*C*). PRMT1 inhibition in HeLa cells is known to increase the number of SGs per cell *via* RGG/RG motif methylation of G3BP1 (50) and UBAP2L (59). To demonstrate the role of arginine methylation suppression of G3BP1 SGs was due to N protein methylation, *per se*, we transfected Huh-7 cells with WT and R-K Flag-N and monitored SGs. Cells with N protein with R95K or R95K/R177K substitution showed increased SG formation in comparison with those transfected with Flag-N or Flag-N R177K (Fig. 4*A*, R95K 35.82 ± 3.03%, R177K 26.74 ± 2.52%, R95K/R177K 36.62 ± 2.78% *versus* WT N 25.30 ± 2.62%). These findings show that the methylation of N protein at R95, but not R177, is required for the SARS-CoV-2 N to suppress G3BP1-positive SGs.

We next examined whether the ectopic expression of a methylarginine reader could also interfere with N protein–mediated regulation of G3BP1 SGs. The Tudor domain is a known reader of methylated arginine residues (34). We first examined whether Flag-N associated with ectopically expressed Tudor domain–containing proteins survival of motor neuron (SMN) and Tudor domain-containing protein 3 (TDRD3) by coimmunoprecipitation assays. Indeed, a strong interaction between Flag-N and TDRD3 was observed, whereas the interaction with SMN was weaker, as visualized by immunoblotting (Fig. 4*B*). Next, we tested whether TDRD3 influenced N protein–mediated SG regulation, as it is known that TDRD3 localizes to SGs (60, 61). We cotransfected increasing amounts of expression plasmid encoding TDRD3 together with Flag-N and visualized the presence of arsenite-induced G3BP1 SGs. We observed an increase in G3BP1 SGs with increased expression of TDRD3 with N protein (Fig. 4*C*). These findings suggest that increasing the methyl reader TDRD3 expression could be a means to quench the effects of N protein on SG regulation.

### Arginine methylation of R95 and R177 is required for N protein binding to the SARS-CoV-2 5’-UTR RNA

**Figure 5:**
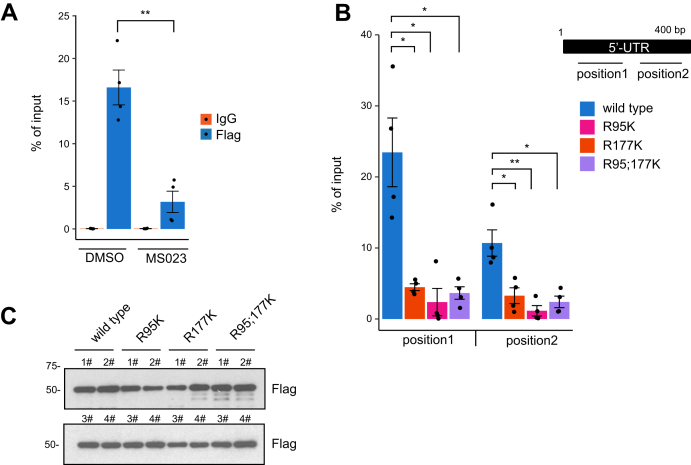
**Arginine methylation of N R95 and R177 is a requirement for SARS-CoV-2 N binding to the 5’-UTR of its genomic RNA.***A*, HEK293 cells were cotransfected with the plasmid expressing Flag-N and the plasmid expressing a 400 bp RNA fragment of the SARS-CoV-2 5’-UTR region (p5’-UTR:CoV-2, NC_045512: 1–400 bp). The cells were incubated with 5 μM MS023 or DMSO for 24 h. Cells were cross-linked with 1% formaldehyde and subjected to RIP using IgG or anti-Flag antibodies. The immunoprecipitated RNA was extracted, and RT-qPCR was used to assess the bound RNA. Data are shown as the percentage of input from two independent experiments. Welch's *t* test. ∗∗*p* < 0.01. *B* and *C*, HEK293 cells were cotransfected with plasmids expressing WT and RK N proteins along with the plasmid expressing p5’-UTR:CoV-2 RNA. Then, the cells were treated with 4-thiouridine for 16 h and subjected to PAR-CLIP analysis (*B*). RT-qPCR with primers targeting consecutive regions, shown in the diagram, were used to assess the bound RNA. Data are shown as the percentage of input from two independent experiments. Welch's *t* test. ∗*p* < 0.05, ∗∗*p* < 0.01. Immunoblotting for expression of indicated proteins from two independent experiments is shown, respectively, in the *top* and *bottom panels* (*C*). DMSO, dimethylsulfoxide; IgG, immunoglobulin G; PAR-CLIP, photoactivatable ribonucleoside–enhanced crosslinking and immunoprecipitation; RIP, RNA immunoprecipitation; SARS-CoV-2, severe acute respiratory syndrome coronavirus 2.

N is an RNA-binding protein that binds the 5’-UTR of its viral genomic RNA for viral ribonucleoprotein (vRNP) formation and packaging into virions (62, 63). The SARS-CoV-2 N protein R95 and R177 are located in the NTD and at NTD–SR linker boundary, respectively. Actually, R95 and R177 are within the RNA-binding site of the NTD with R177 being predicted to be implicated in N protein RNA binding (4, 11, 64). Therefore, we reasoned that these arginines and their methylation were likely involved in the RNA-binding activity of N protein. HEK293 cells were cotransfected with Flag-N and an expression vector transcribing ∼400 bp of the 5’-UTR RNA sequence of SARS-CoV-2 (p5’-UTR:CoV-2; Fig. 5*A*). Initially, we performed RNA immunoprecipitation (RIP) to monitor N protein RNA-binding activity. We observed a >5-fold enrichment with anti-Flag antibodies in the DMSO-treated cells *versus* MS023-treated cells (Fig. 5*A*). These data suggest that inhibition of type I PRMTs prevents the binding of Flag-N to the 5’-UTR of SARS-CoV-2 RNA. Next, we wished to confirm that this N protein/RNA interaction was direct by performing a photoactivatable ribonucleoside–enhanced crosslinking and immunoprecipitation (PAR-CLIP) assay (65). Cells transfected with Flag-N or Flag-N harboring R95K, R177K, R95K/R177K, and p5’-UTR:CoV-2 were labeled with 4-thiouridine and UV cross-linked. The cells were lysed and immunoprecipitated with anti-Flag antibodies after a ‘clipping’ step with RNase A. RNA was purified and analyzed by RT-qPCR with two sets of primers against the 5’-UTR of the SARS-CoV-2 RNA (positions #1 and #2). Using this strategy, we showed that Flag-N directly binds to the 5’-UTR of SARS-CoV-2 RNA (Fig. 5*B*). Importantly, both the single R95K and R177K or the double R95K/R177K substitution of N protein abolished RNA-binding activity (Fig. 5*B*). Immunoblotting was performed to confirm an equal expression of WT N protein and the R-K proteins immunoprecipitated of the four replicates (Fig. 5*C*). Taken together, these results suggest that arginine methylation of both R95 and R177 of the N protein is a requirement for association with its viral RNA *in cellulo*.

### Methylation of N protein is required for SARS-CoV-2 production

**Figure 6:**
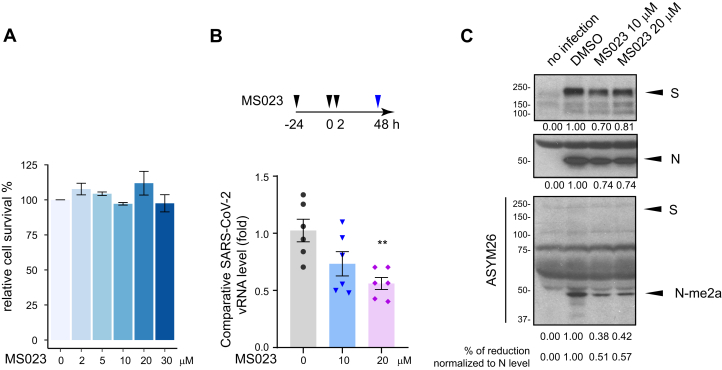
**SARS-CoV-2 replication is impaired by type I PRMT inhibitor MS023.***A*, VeroE6 cells were treated with the indicated dose of MS023 for 3 days as described in the “Experimental procedures” section and cell viability was determined by MTT assay. Data represent the percentage of survival compared with the control (DMSO alone) from three independent experiments. *B*, VeroE6 cells were pretreated with MS023 or vehicle DMSO as control for 24 h. Then, the cells were infected with SARS-CoV-2 at an MOI of 0.1. Two hours later, the medium was refreshed, and cells were incubated with the same concentration of MS023 or DMSO, respectively. Forty-eight hours later, the cell medium containing the released SARS-CoV-2 virions was collected and lysed in TRIzol immediately. Viral RNA was isolated, and TaqMan probe base RT-qPCR was used to assess the viral load. Data represent the fold change against control samples from two independent experiments. Welch's *t* test. ∗∗*p* < 0.01. *C*, viral proteins were extracted from the organic phase of samples in panel *B* and immunoblotted with anti-S, anti-N, and anti-ASYM26 antibodies. The density of the protein bands was calculated using ImageJ. DMSO, dimethylsulfoxide; MOI, multiplicity of infection; PRMT, protein arginine methyltransferase; SARS-CoV-2, severe acute respiratory syndrome coronavirus 2.

We then studied the effect of type I PRMT inhibitor MS0233 on SARS-CoV-2 replication. First, we performed MS023 toxicity assays with VeroE6 cells, a cell line highly susceptible to SARS-CoV-2 infection. We confirmed that cell proliferation was not affected at concentrations of MS023 up to 30 μM in complete medium (Fig. 6*A*). Next, in a certified SARS-CoV-2 BL3 laboratory, we then treated VeroE6 cells with 10 μM or 20 μM MS023, *versus* DMSO control, for 24 h and proceeded with SARS-CoV-2 infection at a low multiplicity of infection (0.1). The infected cells were kept in MS023-containing medium for another 2 days. Supernatant from each, infected well was collected, and the virus inactivated with TRIzol to assay SARS-CoV-2 titer by TaqMan real-time PCR assay. We observed a significant reduction of viral titer when the cells were incubated with 20 μM MS023, and an intermediate viral titer was observed with 10 μM MS023 (Fig. 6*B*).

From the TRIzol organic phase, we extracted the viral proteins and separated them by SDS-PAGE followed by immunoblotting with anti-SARS-CoV-2 S, and N protein or anti-methylarginine ASYM26 antibodies. We observed a slight decrease (∼20–30%) in the total amounts of S and N proteins with MS023 treatment (Fig. 6*C*), consistent with overall decrease in virions (Fig. 6*B*). Notably, the anti-ASYM26 antibodies revealed a ∼50% reduction in N protein arginine methylation, when normalized to N protein levels (Fig. 6*C*, middle panel). In contrast, we could not detect methylation of S with ASYM26 antibody (Fig. 6*C*), likely because of the lack of RGG/RG motifs in S protein sequence. These findings show that the SARS-CoV-2 production is reduced in the presence of type I PRMT inhibitors and that the SARS-CoV-2 N protein is arginine-methylated within the virions.

## Discussion

In the present article, we identify SARS-CoV-2 N protein to be arginine-methylated within virions. We show that methylation of the N protein is mediated by PRMT1 at R95 within the NTD and R177 at the NTD/SR linker boundary. Both residues are within RGG/RG motifs conserved between SARS-CoV and SARS-CoV-2. Amino acid substitutions R95K or R177K inhibited N protein RNA binding in cellulo to SARS-CoV-2 5’-UTR genomic RNA using a CLIP assay in HEK293 cells. The ectopic expression of N protein in Huh-7 cells was localized to cytoplasmic granules and inhibition of G3BP1 SG formation was observed. Notably, arginine methylation of N protein at R95 by PRMT1 was necessary for this function. The N protein interactome was defined to contain many known PRMT1 substrates with RGG/RG motifs. Treatment with the type I PRMT inhibitor MS023 did not influence the overall N interactome, but PRMT1 was identified as a new interactor, consistent with a substrate–enzyme interaction in the presence of the noncompetitive inhibitor MS023 (37). Importantly, inhibition of arginine methylation with MS023 significantly reduced SARS-CoV-2 replication in VeroE6 cells. Our findings define arginine methylation as new mode of interfering with N protein regulation of SGs and define PRMT1 as a requirement for the SARS-CoV-2 life cycle. As type I PRMT inhibitors are in clinical trials for cancer treatment (41), these compounds may also be useful to target SARS-CoV-2 replication.

Host proteins and enzymes are needed for the replication of SARS-CoV-2, and many of these were identified using CRISPR screens (58, 66, 67, 68, 69). Factors required for early viral entry and fusion, and components of the endosome and cholesterol pathways were identified (66, 67, 68). Few components, however, were identified that target the later phases of the viral life cycle, such as vRNP formation, phase separation for genome packaging, encapsidation, and assembly of virions. A recent study identified ∼50 host RBPs binding SARS-CoV-2 genomic RNA and the knockdown of some of these RBPs inhibited viral replication (70). As SARS-CoV-2 N protein and cellular RBPs are substrates of PRMT1 (31), our findings suggest that type I PRMT inhibitors or increasing methylarginine readers may regulate N function in vRNP packaging and assembly into virions. Notably, the increased expression of PRMT1 in nasopharyngeal and bronchial samples of SARS-CoV-2-infected patients (71) is consistent with PRMT1 being a host factor required for the virus.

SARS-CoV-2 viral protein extracts immunoblotted with ASYM26 revealed the arginine methylation of the N protein. It is known that the N protein is highly immunogenic and anti-N antibodies are among the first to appear in infected individuals (72). It is likely that SARS-CoV-2 N protein is fully methylated in infected cells, thus epitopes from the methylated RGG/RG peptides of N protein likely contribute to its elevated immunogenicity. Notably, R95- and R177-derived SARS-CoV-2 N peptides are predicted to be B cell epitopes (73, 74). Moreover, R95 and R177 peptides from the N protein were used to design SARS-CoV-2 vaccines in India (73), and we propose that the incorporation of asymmetrical dimethylarginines would increase their immunogenicity. Although our MS significantly covered 74% of the N protein, the R95 was not covered and R177 was covered, but the peptides were unmethylated. Interestingly, the only variant in N protein is R203K;G204R in the B.1.17 strain and T205I in B.1.351 strain, suggesting the RGT sequence is an important regulatory site. The absence of methylation of the S protein observed using anti-ASYM26 is likely due to the lack of RGG/RG motifs. However, the S protein harbors an RRXR sequence within its furin cleavage site, a potential methylation sequence for PRMT7 (75). Thus, MS analysis and various anti-methylarginine antibodies targeting *N*^*G*^-monomethylarginine and symmetric *N*^*G*^, *N'*^*G*^ dimethylarginine will be needed to further define the sites of arginine methylation within the SARS-CoV-2 viral proteins.

Coronaviruses, like other viruses, have devised strategies to destabilize and inhibit SG formation to ensure optimal viral replication. The infectious bronchitis coronavirus uses endoribonuclease Nsp15 for SG interference (76). For MERS-CoV, it is the 4a accessory protein that inhibits SG formation (77, 78). The N protein from SARS-CoV and SARS-CoV-2 were shown to localize to SGs (14, 21, 26, 27, 28). Thus, targeting the N protein function in G3BP1 SG regulation represents a new and valid strategy to fight COVID-19. It is known that the SR linker region when phosphorylated renders the N protein condensate more liquid-like, and inhibition of N protein phosphorylation favors its translocation to SGs (14, 18, 24, 29, 30, 79, 80). We now show that arginine methylation of N protein is a post-translational modification that tunes N protein regulation of G3BP1 SGs. Information about how SGs are formed and regulated is emerging and represents a combination of multivalent interactions of protein–protein, protein–RNA, and RNA–RNA interactions (81). How does arginine methylation of N protein regulate SG formation? Both R95K and R177K were defective in RNA binding to the SARS-CoV-2 5’-UTR RNA, and yet, only R95K within the NTD was required for SG regulation. Our observation is in agreement with a recent report that residues 1 to 175 of N protein are sufficient to disrupt SGs (26). Consistent with R95 and R177 being part of the U-shaped β-platform of the RNA-binding domain (4, 11), we show that both residues are needed to bind the 5’-UTR where the putative viral packaging signal of the genomic RNA resides. It is possible that N protein R177 is not needed to associate nonspecifically with host mRNAs, and hence, its substitution to lysine does not influence SGs. Thus, we propose that RGG/RG motif methylation of the N protein affects SGs by modulating interaction with methyl readers and RNA.

The N interactome was not significantly altered in the presence of MS023, but there was an increase in PRMT1 association. We noted that increasing the concentration of a methylarginine reader TDRD3 blocked N protein from suppressing SG formation. Therefore, we propose that MS023 besides inhibiting the activity of PRMT1, also inactivates N protein by increasing its interaction with PRMT1, therefore allowing PRMT1 to become an enzyme-inactive RGG/RG motif reader. A role for arginine methylation of the RGG/RG motif in RBP phase separation is known (32, 36). For example, the RGG/RG motif protein FUS, dysregulated in ALS, undergoes liquid–liquid phase separation in the absence of PRMT1 (82, 83). Thus, arginine methylation is a key regulator of ribonucleoprotein condensation. As type I PRMT GSK3368715 inhibitor is in phase I clinical trials for diffuse large B-cell lymphoma (41), our data suggest that these inhibitors may be an effective strategy to interfere with N protein condensation and influence late stages of the SARS-CoV-2 life cycle.

Arginine methylation influences nearby phosphorylation sites often being antagonistic (36). For example, arginine methylation of the FOXO1 transcription factor at R248 and R250 by PRMT1 prevents AKT phosphorylation at S253, blocking nuclear exclusion of FOXO1 (84). Arginine methylation of cytoplasmic tail of the epidermal growth factor receptor at R1175 by PRMT5 enhances its *trans*-autophosphorylation at Y1173 (85). N protein RNA-binding activity is known to be regulated by phosphorylation. *In vitro* studies showed that hypophosphorylation of the N protein facilitated interaction with RNA (21). We show that methylation of N protein R95 and R177 is needed for RNA binding. As S176, S180, S183, and S184 are reported to be phosphorylated by SRPK1 (29) and GSK3–cyclin-dependent kinase 1 (18, 30, 79), it is likely that there will be an interplay between phosphorylation and methylation especially near R177 for binding to the 5’-UTR of the SARS-CoV-2 RNA. It is likely that optimal binding of N protein to the 5’-UTR of the SARS-CoV-2 RNA will require a balance of methylarginines and phosphoserines. This interplay may also influence interactions with methyl readers including TDRD3 and phosphoreaders such as 14-3-3 proteins, the latter shown to bind the N protein (86).

In sum, our findings suggest that arginine methylation is required for N protein function, and PRMT1 is an essential regulator implicated in SARS-CoV2 life cycle. As PRMT inhibitors are in clinical trials (36), they may have applications for COVID-19, in addition to them being promising cancer drug candidates.

## Experimental procedures

### Reagents and antibodies

Immunoblotting was performed using the following antibodies: mouse anti-Flag monoclonal antibody (F1804, Sigma Aldrich, 1:2000); rabbit anti-SARS-CoV-2 N antibody (1:2000, 9103, Prosci); rabbit anti-SARS-CoV-2 S antibody (1:1000, PA5-81795, Invitrogen); rabbit anti-TDRD3 antibody (1:1000, Bethyl Laboratory); mouse anti-SMN antibody (1:2000, 610646, BD Biosciences); rabbit anti-G3BP1 antibody (1:1000, 1F1, Rhône-Poulenc Rorer, a kind gift from Dr Imed Gallouzi at McGill University) (87); rabbit anti-ASYM26 (1:1000, 13-0011, EpiCypher). Immunofluorescence was performed with the following antibodies: mouse anti-Flag monoclonal antibody (F1804, Sigma Aldrich, 1:500); rabbit anti-G3BP1 antibody (1F1, 1:500). Alexa Fluor–conjugated goat anti-rabbit, goat anti-mouse secondary antibodies were from Invitrogen. Protease inhibitor cocktail and protein phosphatase inhibitor cocktail were from Roche. MS023 (SML1555), sodium arsenite (S7400), Protein A-Sepharose (P3391), and PRMT5:MEP50 active complex (SRP0145) were purchased from Sigma Aldrich. Protein coimmunoprecipitation was performed using ANTI-FLAG M2 Affinity Gel (A2220, Sigma Aldrich).

### Cell culture and transfection

HEK293 and VeroE6 cells were maintained in Dulbecco’s modified Eagle’s medium (DMEM) supplemented with 10% fetal bovine serum (FBS) and grown at 37 °C with 5% CO_2_. Huh-7 cells were maintained in DMEM supplemented with 10% FBS and nonessential amino acid (Gibco) and grown at 37 °C with 5% CO_2_. Cells were transfected with 20 nM siRNA oligonucleotides using Lipofectamine RNAiMAX (Invitrogen) according to the manufacturer's instructions. HEK293 and Huh-7 cells were transfected with plasmid DNAs by standard calcium phosphate precipitation and Lipofectamine 3000 (Invitrogen), respectively.

### Plasmids and siRNAs

The N-terminal Flag-tagged SARS-CoV-2 N plasmid was constructed by inserting a Flag-coding sequence into the pcDNA3.1 (+) vector at the *Hind* III and *Bam* HI sites to generate pcDNA3.1-Flag and then the PCR-amplified cDNA of SARS-CoV-2 N coding region at *Bam* HI and *Xho* I sites of pcDNA3.1-Flag vector. The PCR template DNA is a plasmid with insertion of synthesized DNA expressing SARS-CoV-2 N protein provided by Dr Shan Cen based on the SARS-CoV-2 Wuhan-Hu-1 isolate (GenBank: MN_908947). The plasmids for expressing GST fusion proteins of SARS-CoV-2 N truncated fragments were constructed by inserting PCR-amplified SARS-CoV-2 N cDNA fragments in pGEX-6P1 vector at *Bam* HI and *Sal* I sites. The GST–RGG construct was generated by inserting a DNA fragment expressing the mouse RBMX C-terminal RGG/RG motif in pGEX-6P1 vector at *Bam* HI and *Sal* I sites. The mutants with replacement of the arginine residues with lysine at the RGG/RG motif were constructed by a two-step PCR strategy. p5’-UTR:CoV-2 was constructed using synthesized DNA fragment with the 1 to 400 bp of 5’-UTR of SARS-CoV-2 gRNA (NC_045512), and the DNA fragment was inserted into pcDNA3.1 vector at *Bam* HΙ and *Xba* Ι sites. The myc-tagged SMN and TDRD3 plasmids were generated in previous studies (88, 89). All siRNAs were purchased from Dharmacon. siRNA sequences are as follows: siPRMT1, 5′-CGT CAA AGC CAA CAA GTT AUU-3′. The siRNA 5′-CGU ACG CGG AAU ACU UCG AdTdT-3′, targeting the firefly luciferase (GL2) was used as control. 20 nM siRNA was used for transfection.

### Protein purification and *in vitro* methylation assay

Expression of GST fusion proteins in bacteria was induced with 1 mM IPTG at room temperature (RT) for 16 h. All steps of the purification after growth of bacteria were performed at 4 °C. Cells were lysed by sonication in PBS containing a mixture of protease inhibitors. The lysate was clarified by centrifugation, and the supernatant was incubated with glutathione-Sepharose 4B beads for 2 h. The resin was washed four times with PBS and then twice with 50 mM Tris HCl, pH 7.4, buffer. Protein was eluted with 10 mM reduced glutathione in 50 mM Tris HCl, pH 7.4, buffer. Approximately 10 μg of each GST fusion protein was incubated with 1 μl of (methyl-^3^H) S-adenosyl-L-methionine solution (15 Ci/mmol stock solution, 0.55 μM final concentration, PerkinElmer) and 1 to 2 μg of PRMTs in methylation buffer (50 mM Tris HCl, pH 7.4, 1 mM DTT) for 1 to 4 h at 25 °C or 37 °C. Samples were separated by SDS-PAGE and stained with Coomassie Blue. After destaining, the gel was then incubated for 1 h in EN^3^HANCE (PerkinElmer) according to manufacturer's instructions, and the reaction was visualized by fluorography.

### Cell lysis, immunoprecipitation, and immunoblotting

For coimmunoprecipitation experiments, cells were lysed in the lysis buffer (50 mM Hepes, pH 7.4, 150 mM NaCl, 1% Triton X-100, and a cocktail of protease inhibitors and phosphatase inhibitors). Cell lysates were cleared with high-speed centrifugation to remove cell debris, and then, the supernatant was incubated with anti-Flag M2 beads for 1.5 h at 4 °C. Samples were washed with 1 ml of the lysis buffer for three times and eluted with 2× SDS loading buffer for Western blot analysis. For LC-MS/MS, the beads were further washed with PBS twice. The beads together with the bound proteins were subjected to LC-MS/MS.

### LC-MS/MS analysis

For FLAG-N proteomic analysis, peptides were reconstituted in water containing 0.2% formic acid and analyzed by nanoflow-LC-MS/MS using an Orbitrap Fusion Mass spectrometer (Thermo Fisher Scientific) coupled to a Proxeon Easy-nLC 1000. Samples were injected on a 150 μm, ∼ 20 cm nano-LC column (Jupiter C18, 3 μm, 300 Å, Phenomenex). The separation was performed with a linear gradient from 5 to 30% ACN and 0.2% formic acid over 56 min at 600 nl/min. Full MS scans were acquired from m/z 350 to m/z 1200 at resolution 120,000 at m/z 200, with a target automatic gain control of 5E5 and a maximum injection time of 50 ms. MS/MS scans were acquired in CID mode with a normalized collision energy of 30% at the rapid scan rate using a Top 3s method, with a target automatic gain control of 2E4 and a maximum injection time of 35 ms. Dynamic exclusion was set at 60 s. Database searches were performed with PEAKS X against the UniProt human database (20,350 entries) with the virus sequence. Precursor and fragment tolerances were set at 10 ppm and 0.01 Da, respectively. Carbamidomethylation (C) was selected as fixed modification. Phosphorylation (STY), deamidation (NQ), and oxidation (M) were selected as variable modifications.

### MS023 toxicity assay

Before performing viral infections, the MS023 inhibitor was examined for toxicity to the VeroE6 cells. VeroE6 cells (2500 cells per well) were seeded in a 96-well plate and cultured at a condition similar to that in the viral infection analysis. The type I PRMT inhibitor MS023 was dissolved in DMSO and diluted in complete DMEM containing 10% FBS. The medium was added to cells with a final concentration of 2 to 30 μM MS023 and 0.2% DMSO as indicated. Twenty-four hours later, the cell culture medium was replaced with 2% FBS/DMEM medium containing the same concentration of MS023 and DMSO in the corresponding wells. After 48 h of further incubation, the cell viability was analyzed using the MTT assay kit (Abcam, ab211091) according to manufacturer's instructions. Briefly, the medium was carefully removed, and both 50 μl of serum-free medium and 50 μl of MTT reagent were added to each well. After 3 h of incubation at 37 °C, the MTT reagent was removed and 150 μl of MTT solvent was added to each well and the plate was incubated at RT on an orbital shaker for 30 min before reading absorbance at 590 nm. The absorbance of MS023-treated wells was divided by the absorbance of the DMSO-treated wells to normalize cell survival.

### SARS-CoV-2 infection and purification of genomic SARS CoV-2 RNA (gRNA) and proteins

Within the certified BL3 containment facility of the McGill University Health Centre, VeroE6 cells were seeded in 24-well plates (10^5^/well in 0.5 ml) and incubated in complete DMEM containing 10% FBS in the presence of PRMT1 inhibitor MS023 or DMSO control for 24 h before infection. The cells were then infected with SARS-CoV-2 isolate RIM-1 (GenBank accession number: MW599736) at multiplicity of infection (0.1) at 37 °C for 2 h. The virus inoculum was removed, and the cells were washed once with 2% FBS/DMEM and then incubated for an additional 48 h in 1 ml of 2% FBS/DMEM containing the PRMT inhibitor at indicated concentrations or the same amount of DMSO as control at 37 °C. After the infection was complete, 250-μl cell supernatant was lysed in 0.75 ml TRIzol LS (Invitrogen) and transported out of the BL3 facility. The viral RNA was then extracted from the TRIzol using chloroform extraction following manufacturer's instructions. One-step RT-qPCR was performed using TaqMan Fast Virus 1-Step Master Mix following the manufacturer’s instructions. Viral gRNA was detected using primers (Fw: 5’-ATG AGC TTA GTC CTG TTG-3’, Rv: 5’-CTC CCT TTG TTG TGT TGT-3’) and probe (5’Hex-AGA TGT CTT GTG CTG CCG GTA-BHQ-1-3’), specifically targeting *RdRp* gene as described (90). In addition, viral proteins were extracted from the organic phase of TRIzol solution according to the manufacturer’s instruction. Briefly, after removing the aqueous phase, 0.3 ml 100% ethanol was added to the organic phase. Genomic RNA (gRNA) from infected cells was pelleted by centrifugation at 2000*g* for 5 min. About 0.75 ml of the supernatant was moved to a new tube and mixed with 1.5 ml isopropanol. Proteins were collected by centrifugation at 12,000 rpm for 15 min, followed by two times of washing with 0.3 M guanidine hydrochloride and 95% ethanol. Liquid was removed, and the pellet was air-dried. The dried proteins were dissolved in 1X SDS loading buffer and proceed with Western blot analysis.

### PAR-CLIP and RIP

PAR-CLIP was performed as previously described with minor modification (91). p5’-UTR:CoV-2 and pFlag-N were cotransfected to HEK293 cells with a 1:1 ratio. 24 h after transfection, the cells were treated with 100 μM 4-thiouridine for 16 h and cross-linked with 0.15 J/cm^2^ 365 nm UV. Cells were then washed twice with ice-cold PBS and resuspended in the lysis buffer (150 mM KCl, 25 mM Tris, pH 7.4, 5 mM EDTA, 0.5 mM DTT, 0.5% NP40, and 100 U/ml RNase inhibitor). After incubation for 20 min with rotation at 400 rpm and cell debris were cleared by centrifugation. Cell lysates were incubated with 1 U/ml RNase I at 37 °C for 3 min. For each immunoprecipitation, 40 U RNase inhibitor and 2 μg of antibody was added, and the samples were incubated for 2 h at 4 °C with rotation. Protein A Sepharose beads (Sigma) were then added and the samples were incubated at 4 °C for another hour with rotation. The beads were pelleted by centrifugation, resuspended, and washed in high salt wash buffer for three times and lysis buffer once. After removing the final wash buffer, DNA fragments were digested with 2U TURBO DNase (Thermo fisher, AM2238) at 37 °C for 4 min. RNA was eluted in Proteinase K buffer (50 mM Tris, pH 7.5, 75 mM NaCl, 6.5 mM EDTA, and 1% SDS) supplemented with proteinase K and incubated at 50 °C for 30 min. RNA was recovered by using five volumes of TRIzol LS Reagent (Thermo Fisher). Equal volume of RNA from each sample was used for the reverse transcription. qPCR was performed using primers targeting gRNA 5’-UTR. Primer sequences used in the experiment are as follows: position 1: Fw: 5’-TCG TTG ACA GGA CAC GAG TA-3’, Rv: 5’- CCC GGA CGA AAC CTA GAT GT-3’; position 2: Fw: 5’- CCT TGT CCC TGG TTT CAA CG-3’, Rv: 5’- CAC GTC GCG AAC CTG TAA AA-3’.

RIP was performed as previously described with minor modifications (92). Briefly, cells were cross-linked with a final concentration of 1% formaldehyde, washed twice with ice-cold PBS, and resuspended in RIP buffer (150 mM KCl, 25 mM Tris, pH 7.4, 5 mM EDTA, 0.5 mM DTT, 0.5% NP40, and 100 U/ml RNase inhibitor). Chromatin was sheared by sonication, and DNA fragments were digested with TURBO DNase (Thermo fisher, AM2238) at the 37 °C for 15 min. The cell lysate was proceeded with immunoprecipitation with the antibody and washed with the RIP buffer. The RNA was recovered from the precipitate as described above. qPCR was performed using primers: Fw: 5’-TCG TCT ATC TTC TGC AGG CT-3’, Rv: 5’-ACG TCG CGA ACC TGT AAA AC-3’.

### Arsenite treatment and immunofluorescence

Cells growing on glass coverslips were treated with 0.5 mM arsenite for 1 h and fixed for 10 min with 4% paraformaldehyde (PFA). After three washes with PBS, the cells were permeabilized for 5 min with 0.25% Triton X-100 in PBS. Coverslips were incubated with the blocking buffer containing 5% FBS for 1 h and then incubated with primary antibodies diluted in PBS containing 5% FBS for 2 h. After three washes, the coverslips were incubated with corresponding fluorescent secondary antibodies for another hour in PBS containing 5% FBS. After rinsing, the coverslips were mounted with IMMU-MOUNT (Thermo Scientific) mounting medium containing 1 μg/ml of 4′,6-diamidino-2-phenylindole. Images were taken using a Zeiss LSM800 confocal microscope.

### Statistical analysis

All data are expressed as the mean ± S.E.M. and compared between groups using Welch's *t* test. *p* Value <0.05 was considered statistically significant. ∗*p* < 0.05; ∗∗*p* < 0.01; ∗∗∗*p* < 0.001.

## Data availability

The mass spectrometry proteomics data have been deposited to the ProteomeXchange Consortium (http://proteomecentral.proteomexchange.org) *via* the PRIDE partner repository with the dataset identifier PXD025763.

## Supporting information

This article contains supporting information (47, 93).

## Conflict of interest

The authors declare that they have no conflicts of interest with the contents of this article.
