# Supplementary material for: Arginine methylation of SARS-Cov-2 nucleocapsid protein regulates RNA binding, its ability to suppress stress granule formation, and viral replication
Source: J Biol Chem. 2021 May 23;297(1):100821. doi: 10.1016/j.jbc.2021.100821 (PMC8141346; doi:10.1016/j.jbc.2021.100821)
Supplement: Supplemental Figures S1 and S2 [file mmc2.docx]

**Supplemental Figures**

**
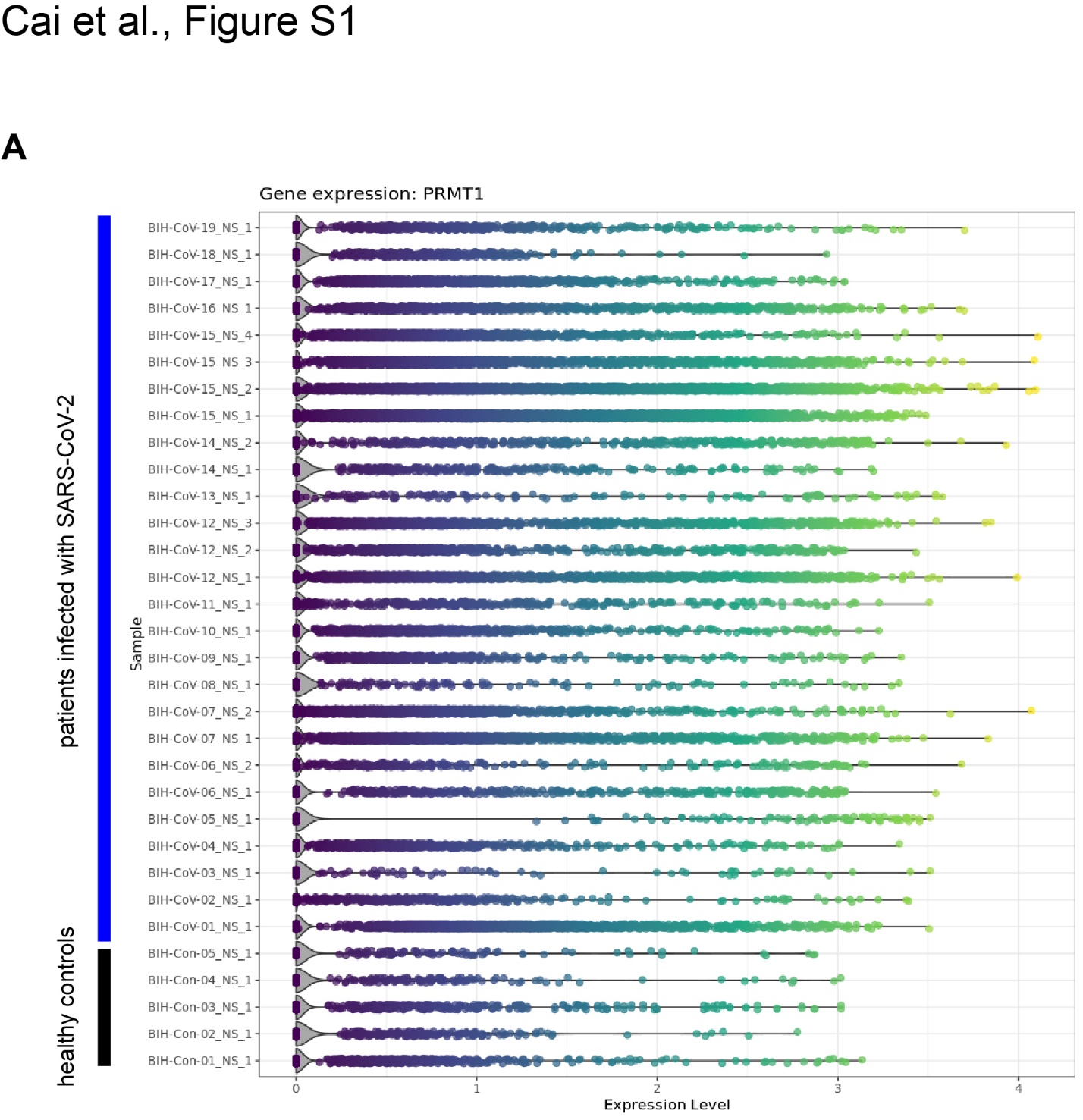
**

**Figure S1. Increased PRMT1 expression in SARS-CoV-2 infected individuals.**

Expression of PRMT1 in single cell RNA seq data generated with nasopharyngeal and bronchial samples from 19 clinically well-characterized patients and five healthy controls

(https://digital.bihealth.org).

**
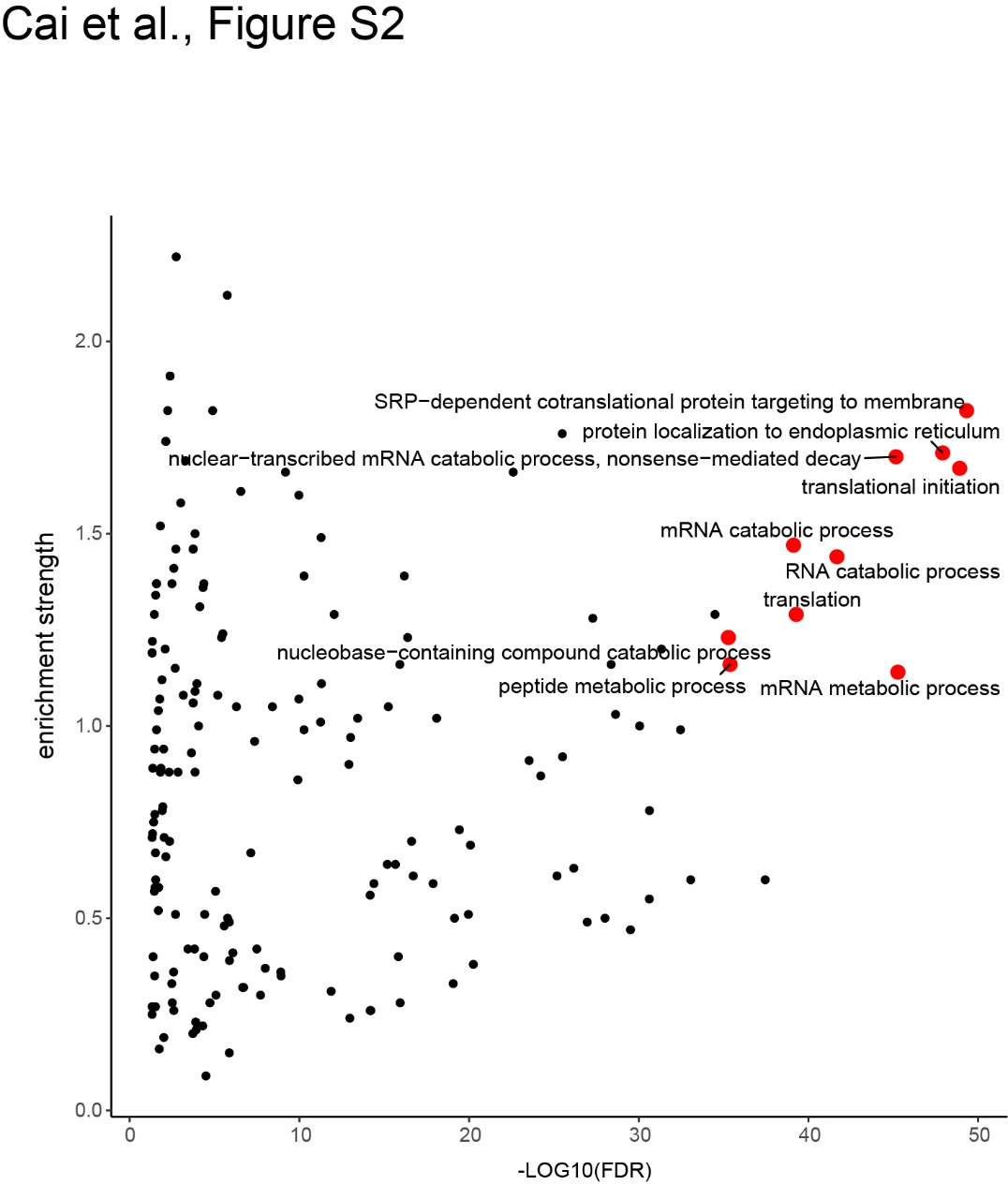
**

**Figure S2. GO analysis of SARS-CoV-2 N protein interactome.**

Biological process (Gene Ontology) was performed using STRING analysis. Significant enriched pathway is highlighted in red and defined as -LOG_10_(FDR) >35 and strength >1.
